# Structural basis of complement membrane attack complex formation

**DOI:** 10.1038/ncomms10587

**Published:** 2016-02-04

**Authors:** Marina Serna, Joanna L. Giles, B. Paul Morgan, Doryen Bubeck

**Affiliations:** 1 Department of Life Sciences, Imperial College London, Sir Ernst Chain Building, South Kensington Campus, London SW7 2AZ, UK; 2 Institute of Infection and Immunity, School of Medicine, Cardiff University, Heath Park, Cardiff CF14 4XN, UK

## Abstract

In response to complement activation, the membrane attack complex (MAC) assembles from fluid-phase proteins to form pores in lipid bilayers. MAC directly lyses pathogens by a ‘multi-hit' mechanism; however, sublytic MAC pores on host cells activate signalling pathways. Previous studies have described the structures of individual MAC components and subcomplexes; however, the molecular details of its assembly and mechanism of action remain unresolved. Here we report the electron cryo-microscopy structure of human MAC at subnanometre resolution. Structural analyses define the stoichiometry of the complete pore and identify a network of interaction interfaces that determine its assembly mechanism. MAC adopts a ‘split-washer' configuration, in contrast to the predicted closed ring observed for perforin and cholesterol-dependent cytolysins. Assembly precursors partially penetrate the lipid bilayer, resulting in an irregular β-barrel pore. Our results demonstrate how differences in symmetric and asymmetric components of the MAC underpin a molecular basis for pore formation and suggest a mechanism of action that extends beyond membrane penetration.

## Abstract (web summary)

The membrane attack complex (MAC) is an immune effector that kills pathogens by forming pores in their membrane. Here the authors use cryo-electron microscopy to reveal that the full MAC is an asymmetric pore with a split-washer configuration and identify a network of interactions that provide a basis for sequential assembly.

## Graphical abstract

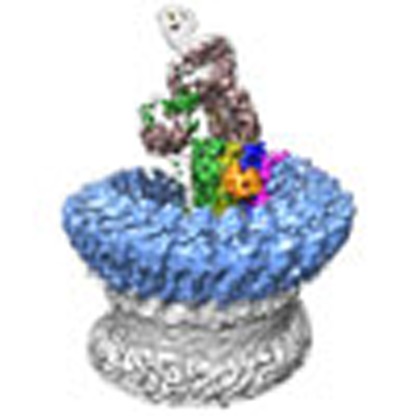

Complement is a phylogenetically ancient component of the innate immune system, providing rapid response to pathogen challenge. Activation of complement triggers assembly of the membrane attack complex (MAC), a multi-protein pore that inserts into and directly lyses microbes^1^. MAC is deployed to kill a wide range of Gram-negative bacteria. It is essential for defense against *Neisseria meningitidis*, with genetic deficiencies in MAC components leading to recurrent infections^2^. Unregulated MAC formation causes host tissue damage^3^, whereas sublytic pore concentrations trigger signal transduction pathways that activate events including proliferation^4^.

MAC assembly requires the sequential and irreversible association of complement proteins C5b, C6, C7, C8 and C9 (ref. 1). The association of cholesterol-dependent cytolysin/MAC/perforin-like (CDC/MACPF) domains, found in all but C5b, is responsible for pore formation. CDC/MACPF proteins comprise a large family of structurally related molecules, many of which form pores involved in host immunity and bacterial pathogenesis. Crystal structures of C6 (ref. 5), C8 (refs 6, 7), perforin^8^ and a number of CDCs^9^,^10^ in their soluble forms define the CDC/MACPF fold as a central kinked β-sheet with three helical subregions CH1, CH2 and CH3. Fluorescence spectroscopy experiments have established that both CH1 and CH2 helical subregions within the bacterial CDC domain unfurl to form lipid-inserted β-hairpins^11^,^12^. Based on structural homology with CDCs, it is predicted that these regions comprise the MAC pore as well.

## Results

### Cryo-EM structure of the MAC

**Figure 1:**
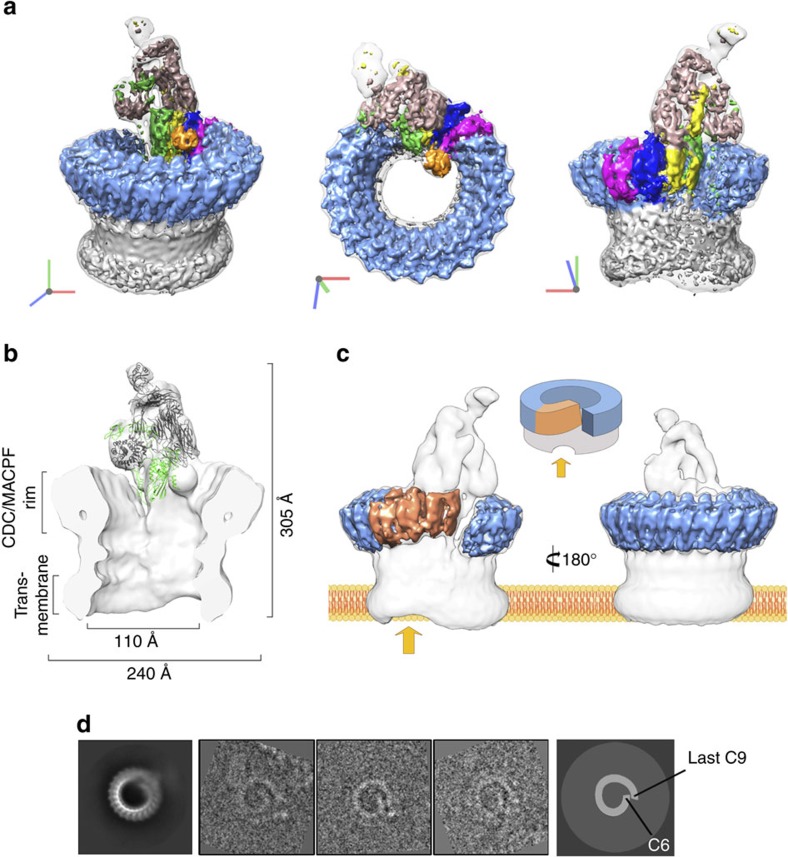
Cryo-EM reconstruction of the MAC. (**a**) Sharpened map segmented and coloured according to complement proteins: C5b (tan), C6 (green), C7 (yellow), C8α (magenta), C8β (dark blue), C8γ (orange) and C9 (light blue). CDC/MACPF β-barrel and detergent belt are in grey colour. Density is overlaid on the unsharpened map (transparent surface). Axes are shown. (**b**) Fitting of the C5b6 crystal structure into a cross-section of the unsharpened map (grey surface). C5b and C6 are in grey colour and green ribbons, respectively. (**c**) ‘Split-washer' configuration of the MAC comprises 4 asymmetric (orange) and 18 symmetric (blue) staves. Incomplete membrane penetration of the β-barrel is indicated (arrow). (**d**) Reference-free aligned class average corresponding to a top view of the MAC (left panel) and representative raw images belonging to this class (middle panel), clearly showing the ‘split-washer' shape of the rim. Cartoon schematic illustrating the location of C6 and the last C9 molecule in the previous images (right panel).

To date, the lack of structural information for MAC has been a major obstacle in deriving a molecular mechanism underpinning its assembly and membrane penetration. We therefore sought to characterize the complete transmembrane pore using electron cryo-microscopy (cryo-EM). Individual complement proteins were purified from human plasma (Supplementary Fig. 1a). MACs were assembled on liposomes and a fluorescent dye-release assay was used to confirm the presence of functional and biologically relevant pores (Supplementary Fig. 1b). Complexes were detergent solubilized and complete MACs were separated from MAC precursors, off-target assembly complexes and irregular arrays probably due to C9 homo-oligomers. Frozen-hydrated samples were imaged in the electron microscope using a direct-electron detection camera (Supplementary Fig. 1c,d). Reference-free aligned class averages based on cryo-EM data were used to generate an initial model for refinement (Supplementary Fig. 2). Individual single particles were subjected to both two-dimensional (2D) and three-dimensional classification procedures to obtain a homogenous population (Supplementary Fig. 2a,b). The structure was refined without symmetry to a final resolution of 8.5 Å, with local resolutions ranging from 6 to 14 Å (Fig. 1 and Supplementary Fig. 3). A soft mask removing flexible regions of the structure was applied during later refinement cycles, improving the resolution to 7.3 Å (Supplementary Fig. 3c,d). The effects of beam-induced motion were corrected by aligning individual frames. A complete model of the MAC was constructed from existing component crystal structures^6^,^13^, nuclear magnetic resonance data for individual domains^14^ and homology models for remaining complement proteins (Supplementary Table 1).

### Molecular architecture of the MAC

The overall structure of the MAC pore exhibits a tubular architecture with a single stalk protrusion (Fig. 1a). The complex measures 305 Å in its longest dimension, while the upper rim of the barrel has an outer diameter of 240 Å. A cross-section of the reconstruction reveals the canonical kinked ‘L'-shape of the CDC/MACPF fold present in the rim of the barrel (Fig. 1b). We observe a cylinder of smooth, continuous density below the CDC/MACPF-rim (Fig. 1c), a feature consistent with a giant β-barrel pore comprising CH1 and CH2 residues. A detergent belt covers the transmembrane region at the base of the barrel, in accord with images of the complex on liposomes^15^. Two rings of density were also visible on the interior of the barrel and could correspond to additional detergent density or glycosylation within the CDC/MACPF.

**Figure 2:**
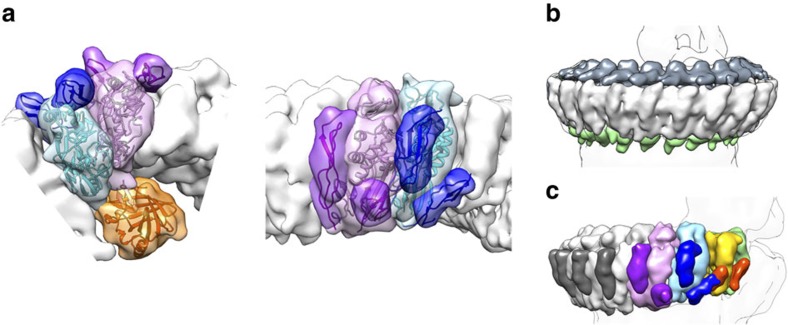
Molecular architecture of the complex. (**a**) Top (left panel) and side (right panel) views of the CDC/MACPF rim zoomed in, to show fitting of C8α (purple), C8β (blue) and C8γ (orange) into the MAC reconstruction (transparent surface). (**b**) Arrangement of LR (dark grey) and EGF (green) domains around the rim. (**c**) Density for the MAC segmented and coloured according to subunit composition: C6 (green), C7 (yellow), C8 (coloured as in **a**) and C9 (grey). TS domains for each protein are indicated by darker shades. Silhouette of the unsharpened map is shown for reference.

**Figure 3:**
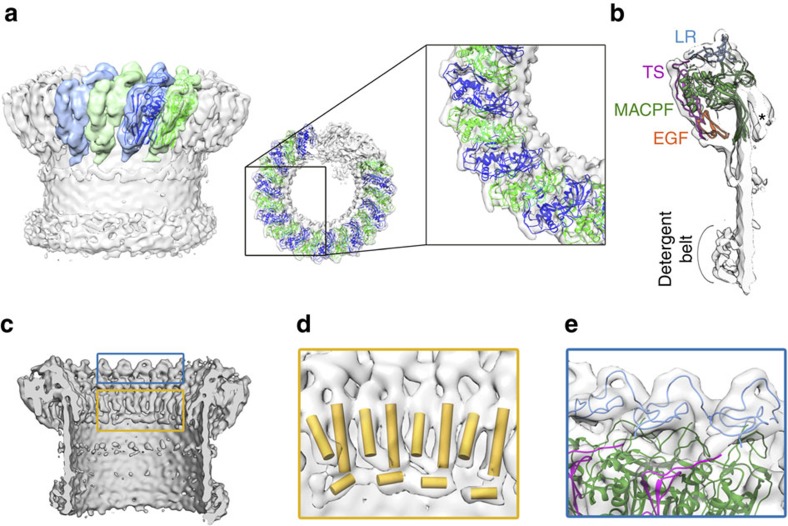
Organization of C9 in the barrel map. (**a**) Two views of the C9 homology model (ribbons) fitted into the reconstruction (grey). Alternating monomers are in green and in blue colour for clarity. (**b**) Slab view showing three sequential C9s (ribbons). Asterisk indicates unmodelled helical density on the concave surface of the rim. (**c**) Cross-section of the reconstruction; regions in **d** and **e** are boxed. (**d**) α-Helical finger-like densities represented as cylinders. (**e**) Close-up of the LR crown.

Although the incorporation of one or more C9 molecules can result in lysis^16^, we determine the stoichiometry of the end product of the complement terminal pathway. The MAC is a hetero-oligomeric pore in which C6, C7, C8α, C8β and C9 each contain a single CDC/MACPF domain (Supplementary Fig. 4). Our structure shows that the barrel of the MAC comprises 22 staves, of which 4 sequential staves differ in tilt and stacking from the remaining 18 (Fig. 1c,d and Supplementary Fig. 5). Docking of the C5b6 crystal structure^13^ into density for the stalk confirms that the complex contains one molecule of C6 and defines its identity as one of the four unique staves (Fig. 1a,b). The globular density for C8γ on the interior of the rim demonstrates that one C8 heterotrimer is present and confirms the orientation of the disulfide-linked C8α CDC/MACPF. Refinement of the C8 crystal structure^6^ localizes two asymmetric staves as C8α and C8β (Figs 1a and 2a). Functional evidence (Supplementary Fig. 1b) and mass spectrometry of purified pores (Supplementary Table 2) confirmed the presence of C7 in the complex; hence, we attribute C7 to the remaining asymmetric stave (Fig. 1a). Antibody labelling of C9 in the fluid-phase form of the MAC (sC5b9)^13^, together with direct binding assays of individual proteins^17^, demonstrates that C9 is the clockwise partner of C8α. As C9 homo-oligomerizes in the MAC, we identify the 18 contiguous symmetric staves as C9 (Figs 1c and 3a, and Supplementary Fig. 5). Previous structural studies of a polymerized C9 homo-oligomer report a range of C9 molecules that comprise a closed ring^18^. Based on 2D class averages of top views, we do not observe stoichiometric heterogeneity within the complete MAC. This difference may be attributed to a specific template geometry established by the asymmetric component of MAC or may reflect differences in resolution between the two structures. Together, these data define the MAC protein composition and establish a CDC-like orientation of complement CDC/MACPF domains in the pore, in contrast to the inside-out model proposed for perforin^8^.

### MAC is an asymmetric pore

**Figure 4:**
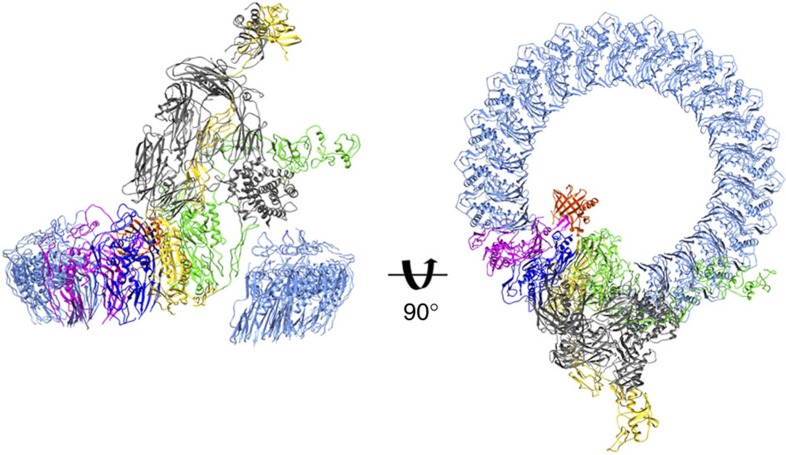
MAC is an asymmetric pore. Pseudo-atomic model of the MAC CDC/MACPF rim, including C5b. Complement proteins coloured as in Fig. 1a.

The most striking feature of the reconstruction is that unlike any other giant β-barrel pore structure solved to date, the MAC is not a symmetric ring. Differences in CDC/MACPF stacking between the symmetric and asymmetric staves give rise to a ‘split-washer' configuration within the rim (Fig. 1c,d), in contrast to the closed ring previously proposed^5^,^6^,^7^,^15^. Our structure shows that the C6 CDC/MACPF juts towards the interior of the barrel (Figs 1,4) where it forms the binding site for C7 and determines the directionality for assembly. C8α CDC/MACPF rotates relative to its position in the closed fluid-phase form, thus aligning the central β-sheets of C8β, C8α and C9 (Supplementary Fig. 6a). This rotation, combined with the concerted movement of C8γ (Supplementary Fig. 6b), opens up the binding site for the first C9 molecule and sets the template geometry that propagates C9 oligomerization (Supplementary Fig. 6c). These data identify a structural role for C8γ within the pore, reconciling functional assays that suggest C8γ facilitates MAC formation, yet its removal does not inhibit lysis^19^. On incorporation into the MAC, the helical CH1 and CH2 regions of C9 (not included in the homology model) convert to their transmembrane β-hairpin form. Indeed, we observe continuous density for the β-barrel cylinder below the core CDC/MACPF domain. Although the C9 homology model lacked well-structured helices for CH3, we observe finger-like densities of α-helices on the concave surface of the barrel that probably occupy the CH3 region (Fig. 3b–d).

### Auxiliary domains and MAC assembly

In addition to the CDC/MACPF, auxiliary domains of complement proteins play key roles in the molecular architecture of the pore. The ‘low density lipoprotein receptor class A repeats' (LR) form a crown adorning the upper surface of the CDC/MACPF rim, while epidermal growth factor (EGF) modules encircle its base (Figs 2b and 3e). In contrast, thrombospondin (TS) domains line the outer surface of the barrel and intercalate between MAC proteins (Fig. 2c). Specifically, we find that the TS1 domain of C7 forms an interface with MAC precursor C5b6, whereas its TS2 module contacts C8β, the next protein in the assembly pathway. The carboxy-terminal TS of C8β interacts with C7, whereas the amino-terminal TS domain of C8α contributes to the binding site for C9. Together, these data provide a molecular basis for interpreting previous functional and biochemical binding assays involving domain deletion mutants^20^. C6 and C7 also possess complement control protein (CCP) and factor I-like (FIM) domains (Supplementary Fig. 4). The crystal structure of C5b6 illustrated how the C6 CCP domains capture and stabilize the labile intermediate, C5b^13^,^21^. This arrangement is preserved in the MAC; however, the C6 FIM domains are flexible and not well resolved (Supplementary Fig. 7a). The CCP domains of C7 line the periphery of the C5b MG scaffold, positioning its two FIM domains proximal to the C345C domain of C5b (Supplementary Fig. 7b). Although this region of the map is the least well ordered due to flexibility of C345C^13^, we observe extra density that could accommodate these domains. This interface is further supported by direct binding studies of C7 FIMs with C345C^22^ and may provide a mechanistic explanation of how C7 binding displaces C5b from the C5 convertase, an interaction also involving C345C^23^.

## Discussion

Our structure defines the molecular architecture of the asymmetric MAC. In contrast to homo-oligomeric perforin and CDC pores, the CDC/MACPF domains of complement proteins comprise a ‘split-washer' configuration within the rim (Figs 1d and 4). It is clear from our density that within the CDC/MACPF rim, C6 and C9 do not make extensive contacts (Figs 1 and 4). The central β-sheets of C6 and C9 are not contiguous in this region, nor does the TS3 domain of C6 form an interface with the final C9 molecule. At the current resolution, we are unable to distinguish strands within the β-barrel or transmembrane region; therefore, we have left this density unmodelled. At the lower resolution of the unsharpened maps, density is continuous for the β-barrel cylinder comprising CH1 and CH2 residues, including both the symmetric and asymmetric staves. These data demonstrate that C6, C7 and C8 form an integral part of the MAC pore and not a peripheral subcomplex, as was extrapolated from polymerized C9 (refs 15, 16, 18). Local resolution estimates in this region reveal that the seam between symmetric and asymmetric regions of the β-barrel is less well ordered (Supplementary Fig. 3). Moreover, we cannot preclude that the MAC may form an arc pore as has been observed for both perforin and CDC homo-oligomers^24^,^25^,^26^.

The transmembrane region of the MAC does not form an idealized β-barrel. Pore-forming hairpins of complement proteins penetrate the lipid bilayer to varying extents. Indeed, lengths of the predicted CH1 hairpins for C6, C7, C8β and C8α are shorter than those for C9 (Supplementary Fig. 8). Our findings are further supported by photolabelling experiments that suggest MAC precursors can insert into, yet not span the bilayer^27^. These data provide a structural basis for how assembly precursors may perturb biophysical properties of the membrane, resulting in a reorganization of the bilayer^28^ and a reduced energy barrier for C9 insertion^29^.

Here we have used single-particle cryo-EM, to determine the structure of the 24-protein human MAC pore (Supplementary Movie 1). The resulting structure suggests a molecular mechanism by which the MAC, in addition to disrupting the membrane, uniquely distorts the lipid bilayer. Indeed, perturbation of membrane curvature has been observed for MACs on liposomes^30^. Deformation of lipid bilayers by membrane proteins is an important component of cell signalling and bacterial mechanosensing, and may also play a part in MAC-mediated lysis and activation of signalling pathways.

## Methods

### Purification of complement proteins and assembled pores

To obtain C5b6, serum (100 ml) depleted of C7 by passage over anti-C7 monoclonal antibody immobilized on sepharose was incubated with zymosan (10 mg ml^−1^) at 37 °C for 2 h, centrifuged to remove zymosan, dialysed against MonoQ buffer (10 mM NaP pH 7.6, 20 mM NaCl), applied to a monoQ column equilibrated in the same buffer and eluted in a salt gradient to 0.5 M NaCl. Fractions were screened for capacity to cause haemolysis of sheep erythrocytes in a reactive lysis assay with added C7, C8 and C9. C5b6 containing fractions were pooled, concentrated to >1 mg ml^−1^ and applied to a Superose 6 gel filtration column equilibrated into Veronal-buffered saline (VBS^2+^), 2.8 mm barbituric acid, 145.5 mm NaCl, 0.8 mm MgCl_2_, 0.3 mm CaCl_2_, 0.9 mm sodium barbital pH 7.2 (Oxoid Ltd). Fractions were screened for C5b6 haemolytic activity, active fractions conservatively pooled, concentrated to >1 mg ml^−1^ and stored in aliquots at −80 °C. C7 was harvested from the immunodepletion column by washing in 20 mM diethylamine pH 11.5, immediately neutralized to pH 7 and dialysed into VBS^2+^ buffer. C8 and C9 were purified from serum by passage over specific monoclonal antibody immobilized on sepharose, eluted and dialysed as for C7. Purity of all proteins was confirmed by SDS–PAGE and Coomassie blue staining; protein concentrations were confirmed using the Bradford assay.

A lipid mixture containing 60% DOPC (Avanti Polar Lipids) and 40% DOPE (Avanti Polar Lipids) were dried down under N_2_ gas, rehydrated in 20 mM HEPES-NaOH pH 7.4, 150 mM NaCl and extruded through membranes with a pore size of 0.1 μm (Avanti Polar Lipids). Individual complement proteins at molar ratios of 1:1:1:18, C5b6:C7:C8:C9, respectively, were incubated with membranes at 37 °C for 1 h and kept overnight at 4 °C. MAC pores were solubilized in 1.5% CyMAL-5 (Anatrace) with 1 mg ml^−1^ DOPC and 10% glycerol for 1 h at room temperature and subjected to density centrifugation through a sucrose solution containing 0.004% CyMAL-7 NG (Anatrace). Mass spectrometry was used to confirm the presence of all MAC proteins in the detergent-solubilized purified pores.

### Mass spectrometry

Samples were analysed by liquid chromatography–mass spectrometry using a nanoAcquity UPLC system (Waters MS Technologies, Manchester, UK). One microlitre of sample was injected onto the trapping column (Waters, C18, 180 μm × 20 mm), using partial loop injection, for 1 min at a flow rate of 15 μl min^−1^ with 0.1% (v/v) formic acid. The sample was resolved on the analytical column (Waters, nanoACQUITY UPLC M-class HSS T3, 75 μm × 150 mm, 1.8 μm column) using a gradient of 97% A (0.1% (v/v) formic acid) 3% B (99.9% acetonitrile 0.1% (v/v) formic acid) to 60% A 40% B over 36 min at a flow rate of 300 nl min^−1^. The nanoAcquity UPLC was coupled to a Synapt G2 mass spectrometer (Waters) and data acquired using a MSE programme with 1-s scan times and a collision energy ramp of 15–40 eV for elevated energy scans. The mass spectrometer was calibrated before use and throughout the analytical run at 1 min intervals using the NanoLockSpray source with glufibrinopeptide. Peptide identification was performed by using ProteinLynx Global SERVER v3.1 (Waters). The data were processed using a low energy threshold of 150 and an elevated energy threshold of 30. A fixed carbamidomethyl modification for cysteine was specified. The search thresholds used were as follows: minimum fragment ion matches per peptide 3, minimum fragment ion matches per protein 7, minimum peptides per protein 2 and a false positive value of 4.

### Fluorescent dye-release assay

Fluorescently labelled liposomes were generated by in the inclusion of 50 mM calcein (Sigma-Aldrich) in the rehydration buffer. This initial calcein concentration of 50 mM is sufficient to cause almost complete self-quenching of its fluorescence; if calcein is subsequently released from the liposomes, its concentration is reduced and an increase in fluorescence is observed. Unencapsulated dye was removed by passage through a G-50 Sephadex column (Sigma-Aldrich) run in a buffer containing 500 mM sucrose. Pore-formation assays were performed at 20 °C in a Cary Eclipse Fluorescence Spectrophotometer (Varian) with excitation/emission wavelengths of 490 nm/520 nm, respectively, and a slit width of 5 nm. The kinetics mode of the spectrophotometer was used with an average read time of 0.15 s and measurements were made every minute. The average background fluorescence intensity of 180 μl of fluorescently labelled liposomes was measured for 10 min. Subsequently, MAC components were added at final concentrations of 0.01 μM C5b6:0.01 μM C7:0.01 μM C8:0.2 μM C9 or 0.01 μM C7:0.01 μM C8:0.2 μM C9. Fluorescence was measured for 60 min. Liposomes were burst at the end of the experiment by the addition of 1 μl 0.2 M C12E8 detergent (Sigma-Aldrich) and the maximum fluorescence was found by monitoring the sample for a further 10 min. Fluorescence measurements for each reaction were normalized according to the background and detergent readings.

### Negative stain electron microscopy

The presence and homogeneity of the complete MAC in each fraction was assessed by negative stain electron microscopy. A volume of 2.5 μl of MAC was applied to glow-discharged carbon-coated copper grids (Electron Microscopy Sciences). Grids were negatively stained with 2% uranyl acetate. Images were taken under low-dose conditions at a nominal magnification of 52,000 on a Tecnai 12 electron microscope (FEI) operated at 120 kV. Images were recorded on a 2 k × 2 k TemCAM-F216 CMOS camera (TVIPS) at 2.49 Å per pixel.

### Electron cryo-microscopy and image processing

Aliquots (2.5 μl) of purified MAC were applied to glow-discharged holey carbon grids (QUANTIFOIL R 1.2/1.3 or QUANTIFOIL R 2/2) in which an additional thin carbon film has been deposited. Samples were vitrified in liquid ethane using a Vitrobot (FEI) and stored at liquid nitrogen temperature. For the initial model, 70 micrographs were acquired on a Tecnai F20 electron microscope (FEI) operated at 200 kV using a Falcon II direct electron detector (FEI) with a defocus range of 2.5–4.5 μm underfocus and at a nominal magnification of 50,000, corresponding to 2.05 Å per pixel. Windowed particles (1,921) were subjected to reference-free alignment using RELION^31^ and classified into 12 classes. The seven best averages were used to obtain an initial template for refinement in the standard EMAN2 initial model generation programme (e2initialmodel.py)^32^. To rule out any potential for model bias due to a possibly incorrect starting model, we also generated a second initial model by merging density for a completely closed symmetric cylinder with a spherical Gaussian blob using Bsoft^33^. Both starting models were strongly low-pass filtered before initiating refinements, which converged on similar reconstructions (Supplementary Fig. 2).

For the refinement, micrographs comprising 33 frames were acquired on a Titan Krios electron microscope (FEI) operated at 300 kV. Six hundred and twenty-two images were recorded with a defocus range of 2–4 μm underfocus and at a nominal magnification of 59,000 on a Falcon II direct electron detector (FEI), corresponding to 1.4 Å per pixel. Images were recorded with a 2-s exposure time, resulting in a total accumulated dose of 45 electrons per pixel.

The first 32 frames were aligned with MotionCorr^34^ and CTF parameters of aligned stacks were estimated using CTFFIND3 (ref. 35). The initial model was filtered to 60 Å and the RELION workflow was followed for the gold-standard refinement^31^. For the MAC reconstruction, images were binned by a factor 2 resulting in a pixel size of 2.8 Å per pixel. Single particles (44,698) were manually picked and subjected to iterative rounds of 2D classification. Following an initial round of refinement, three-dimensional classification was performed to remove images of particles that also contained density for neighbouring particles or ice contaminants. Particles (41,981) were included in the final refinement of the MAC structure. Modulation transfer function correction and B-factor sharpening^36^ were carried using the ‘post-processing' protocols as implemented in RELION. Effects of masking during post processing were accounted for^37^ and the overall resolution was determined. Local resolution estimates were assessed with ResMap^38^. As the resolution of the barrel was considerably better than that of the stalk protrusion, a soft mask removing flexible regions of the structure was implemented in subsequent refinement cycles, as described previously^39^. Unbinned images, corresponding to 1.4 Å per pixel, were subjected to further rounds of 2D classification. A subset of 25,343 particles were included in this focused refinement strategy for the barrel reconstruction.

### Model building and refinement

A model for the MAC was constructed based on crystal structures for C8 (PDB ID: 3OJY) and C5b6 (PDB ID: 4A5W), together with NMR data for the FIM domains of C7 (PDB ID: 2WCY). Homology models for C9 and the remainder of C7 were built with I-Tasser^40^ using the crystal structures of C8α (PDB ID: 3OJY) and C6 (PDB ID: 3T5O), respectively, as templates. The resolution of the maps prevented interpreting the β-hairpin transition of predicted transmembrane segments (CH1 and CH2); therefore, these residues were removed from all complement proteins. CH3 regions of homology models were not well structured and were also left unmodelled. All fitting and automated rigid-body refinement was performed using the sequential fit programme in CHIMERA^41^. Resulting correlation coefficients are reported in Supplementary Table 1. Copies of the C9 model were refined into 16 out of 18 sequential symmetric staves of the sharpened barrel map. The resulting LR domain, as determined by the homology model, was slightly out of density; therefore, we refined C9 as two rigid bodies: the LR domain alone and the second as the remaining C9 model. C9 was regrouped as a single rigid body for all further refinement cycles. Density for the final two staves at the end of the ‘split washer' was less well resolved. To complete the model for the C9 oligomer, the same arrangement was extended by the superposition of molecules from a better-resolved region of the map. Coordinates for C8α, C8β and C8γ were refined in the sharpened MAC map, taking into consideration the position of poly-C9 as defined in the barrel map. C8α and C8β were each split into two rigid bodies, based on predicted hinge points of the structure^5^. One grouping comprises the N- and C-terminal TS domains together with the EGF, LR and linchpin helix of the CDC/MACPF; the remainder of the CDC/MACPF domain was treated as a second rigid body. Density for the stalk protrusion and the final two asymmetric staves was less well resolved in the sharpened MAC map; hence, the core of C7 (TSs, LR, CDC/MACPF and EGF), C7 CCPs and crystal structure of C5b6 were refined as three rigid bodies in the unsharpened map, taking into consideration the positions of C8 and C9 oligomer as defined previously. Handedness of the reconstruction was identified by the docking of the chiral C5b6 crystal structure into the stalk protrusion. Flexible regions of C5b6, C5 C345C, C6 TS3 and C6 FIMs, were removed from the refinement. Although density in the stalk prevented the precise orientation of the C5 C345C domain and C7 FIMs, these domains were manually placed into the unsharpened MAC map without refinement to show approximate domain positions.

All figures were rendered using CHIMERA.

## Additional information

**Accession codes:** Cryo-EM density maps have been deposited at the Electron Microscopy Data Bank under accession numbers EMD-3135 and EMD-3134.

**How to cite this article:** Serna, M. *et al*. Structural basis of complement membrane attack complex formation. *Nat. Commun.* 7:10587 doi: 10.1038/ncomms10587 (2016).

## Supplementary Material

Supplementary Information Supplementary Figures 1-8 and Supplementary Tables 1-2

Supplementary Movie 1 MAC reconstruction showing the fitting of component proteins: C5b6, C7, C8, and C9
