# Supplementary material for: Structural basis of complement membrane attack complex formation
Source: Nat Commun. 2016 Feb 4;7:10587. doi: 10.1038/ncomms10587 (PMC4743022; doi:10.1038/ncomms10587)
Supplement: Supplementary Information — Supplementary Figures 1-8 and Supplementary Tables 1-2 [file ncomms10587-s1.pdf]

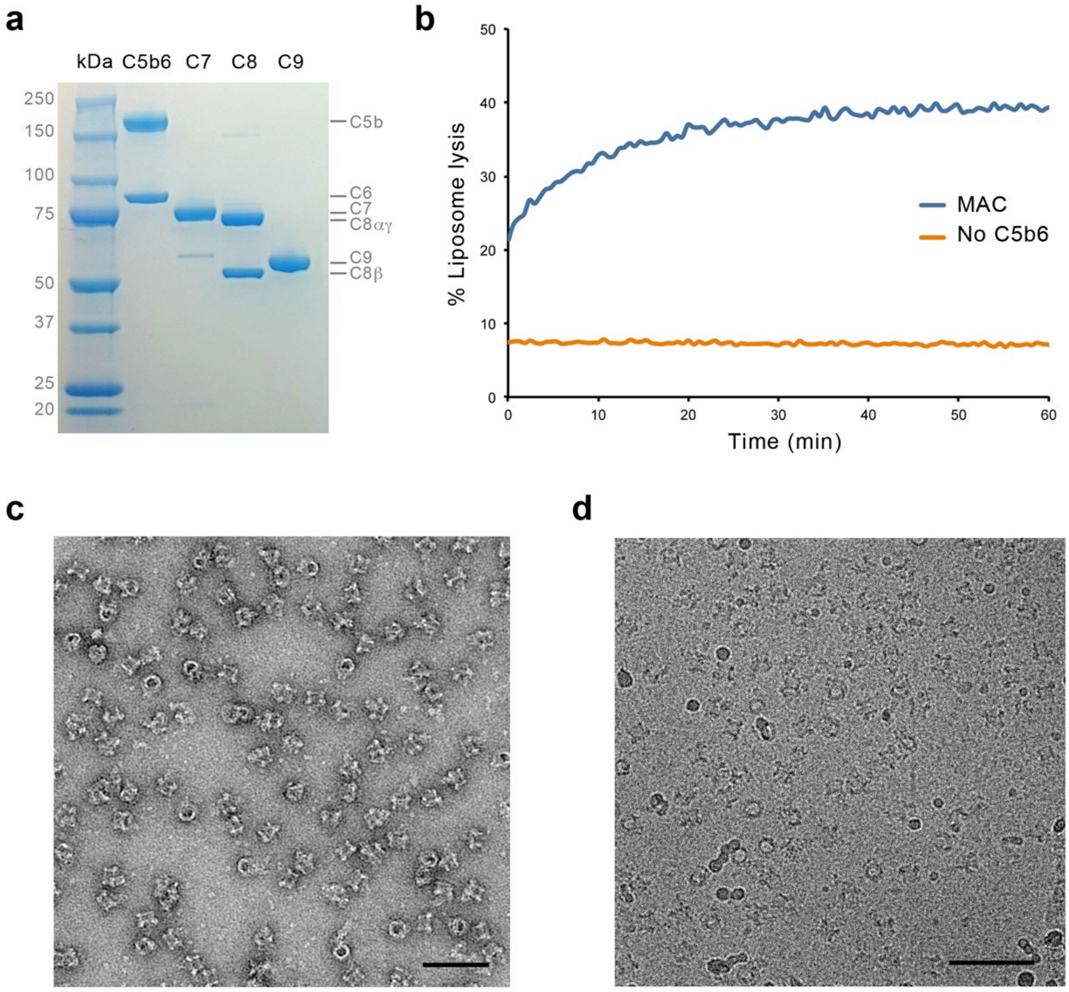

Supplementary Figure 1| MAC purification and functional assay. (a) Coomassie-stained nonreduced SDS-PAGE gel showing purified MAC components. Molecular weight markers are indicated. **(b)** Fluorescent dye-release assay confirms the formation of functional pores on membranes. Calcein-containing liposomes were incubated with a mixture of individual complement proteins: C5b6, C7, C8 and C9 (blue) or C7, C8, and C9 alone (orange). **(c)** Negatively-stained purified pores show that the sample is homogenous, monodisperse, and adopts a range of orientations on the carbon-coated grid. Scale bar, 100nm. **(d)** A representative electron micrograph of the MAC on a carbon-coated quantifoil grid, frozen in vitreous ice. Scale bar, 100nm.

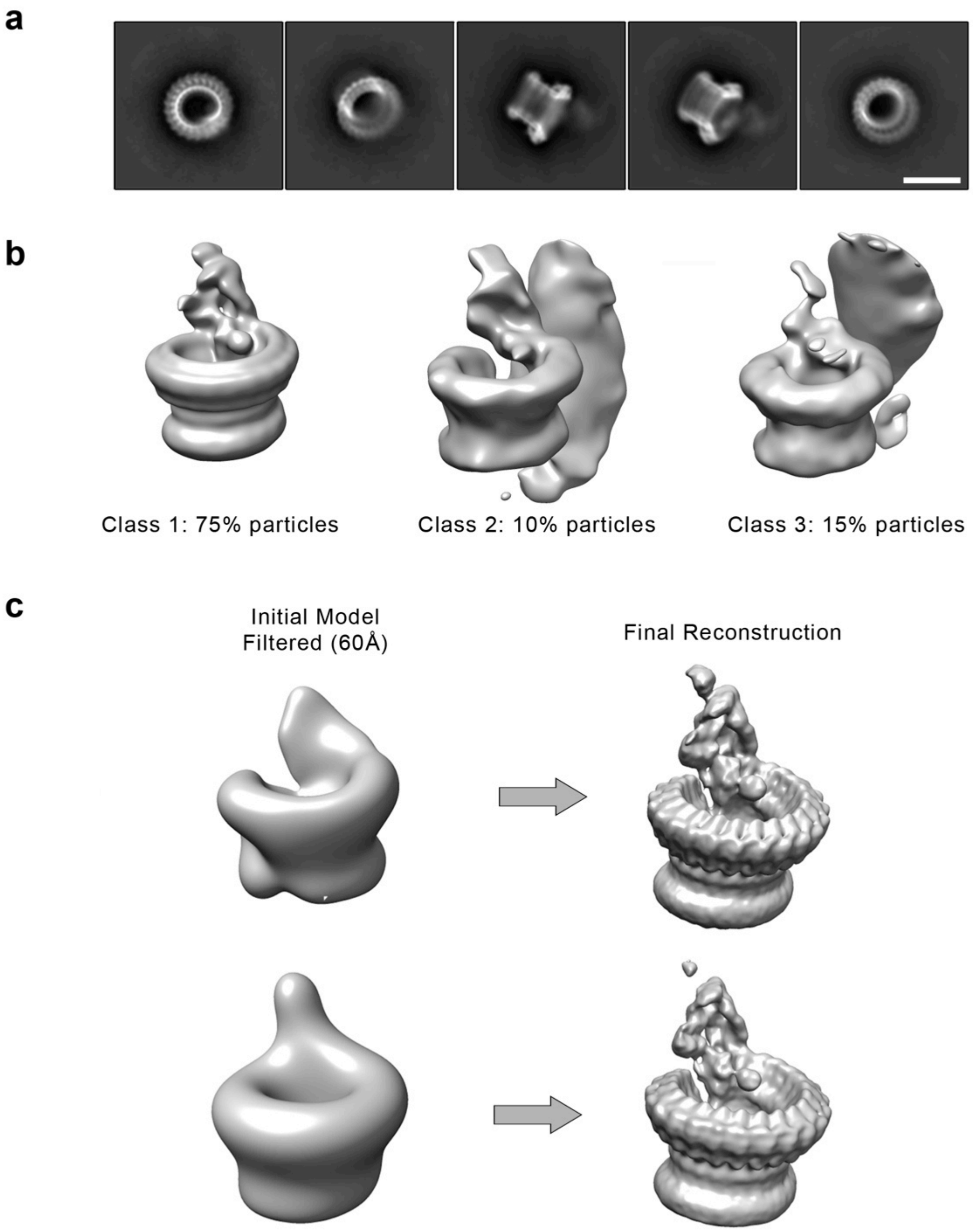

Supplementary Figure 2| Image-processing of the MAC reconstruction. (a) Selected 2D class averages derived from cryo-EM images. Scale bar, 20nm. **(b)** 3D classification separating images of particles that also include density for neighboring particles and ice contaminants. Classes 2 and 3 were removed from further refinement. **(c)** 3D Refinement strategy. An initial model generated in EMAN2, derived from reference-free class averages and filtered to 60 Å (top panel) was used to initiate refinement of the MAC. A second initial model was generated to rule out any bias stemming from a possible incorrect starting reference (bottom panel). The second initial model was generated by merging a symmetric cylinder with a Gaussian blob, which served as a proxy for the asymmetric stalk. The composite model was low-pass filtered to 60 Å and used for an independent refinement, which converged to a similar final reconstruction that also possessed a "split-washer" configuration in the rim.

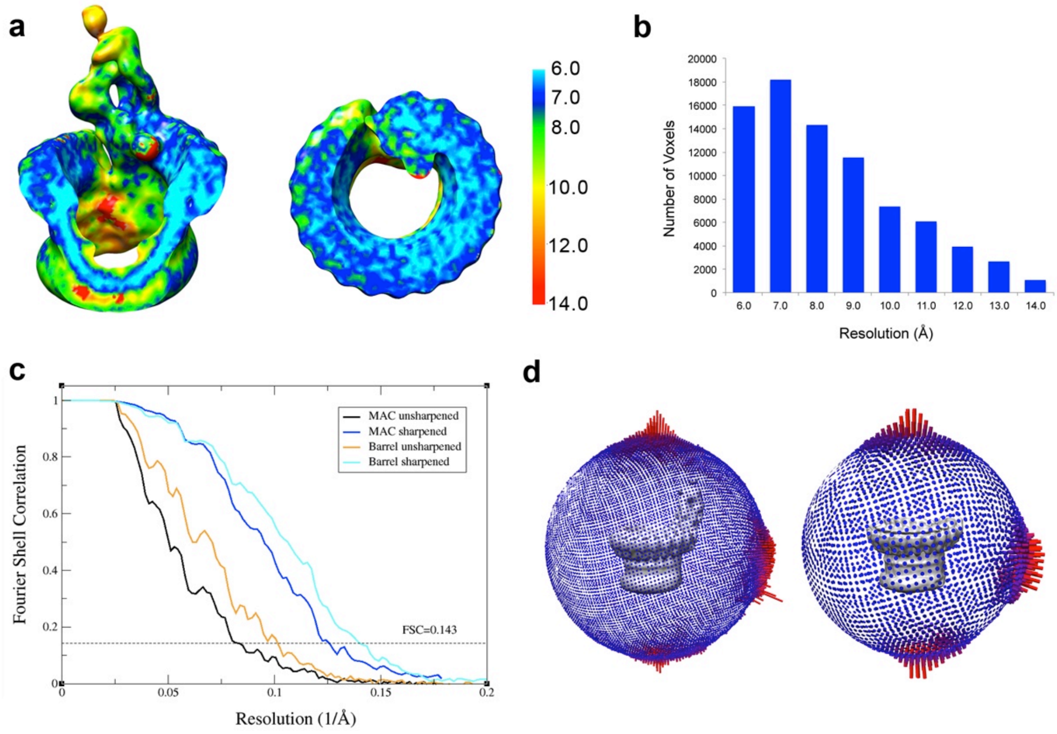

Supplementary Figure 3| Resolution estimation of the reconstructions. (a) 3D density map of the complete MAC (unsharpened and unfiltered) with the local resolution estimated by ResMAP and colored as shown in panel. **(b)** Histogram indicating the distribution of voxels across the resolution spectrum. **(c)** FSC curves for the whole MAC and for the masked barrel, both sharpened and unsharpened. **(d)** Angular distribution for reconstructions of the MAC (left panel) and for the barrel (right panel). Height of the cylinder at each projection direction is proportional to the number of particle images.

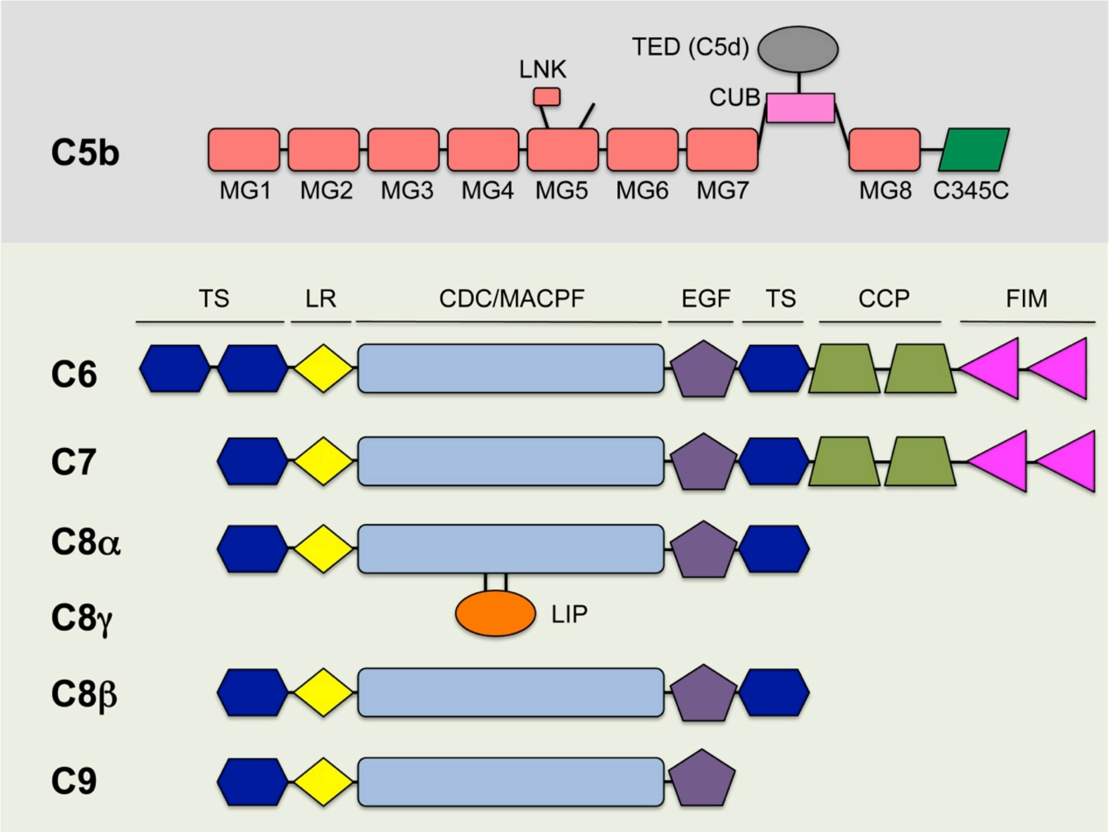

Supplementary Figure 4| Schematic illustrating the domain composition of MAC protein subunits. C5b (top panel) has 8 macroglobulin domains (MG1-8), CUB domain, thioester containing domain (TED), extended linker region (LNK), and C-terminal C345C domain. Thrombospondin (TS), "low density lipoprotein receptor class A repeats" (LR), epidermal growth factor (EGF), cholesterol-dependent cytolysin/membrane attack complex/perforin fold (CDC/MACPF), complement control protein (CCP), and factor I-like modules (FIM) are indicated for the remaining complement proteins (bottom panel). The disulfide bond linking the C8γ lipocalin (LIP) to C8α CDC/MACPF is indicated.

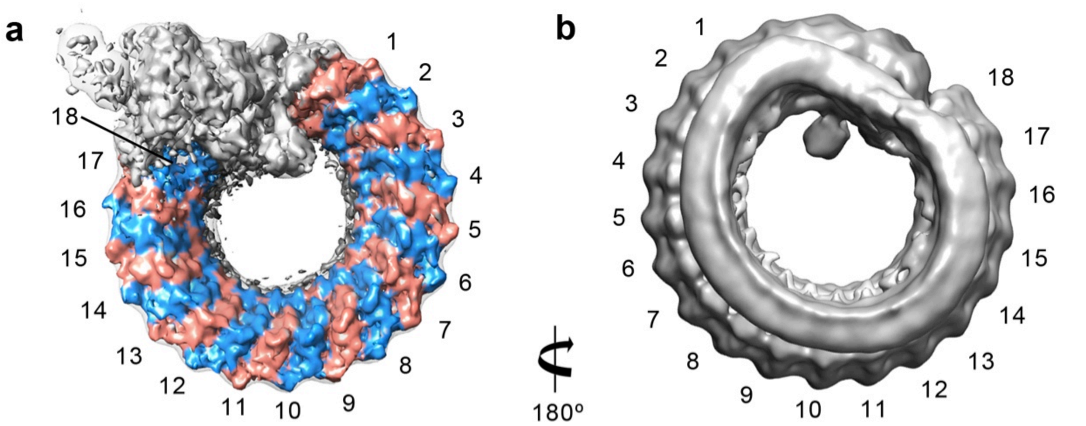

Supplementary Figure 5| Symmetric and asymmetric components of the MAC pore. (a) Top view of the MAC reconstruction in which density is segmented and colored for each of the 18 symmetric staves. Density for the unsharpened map is overlaid (transparent surface). **(b)** Bottom view of the unsharpened reconstruction showing the asymmetric, irregular β-barrel pore.

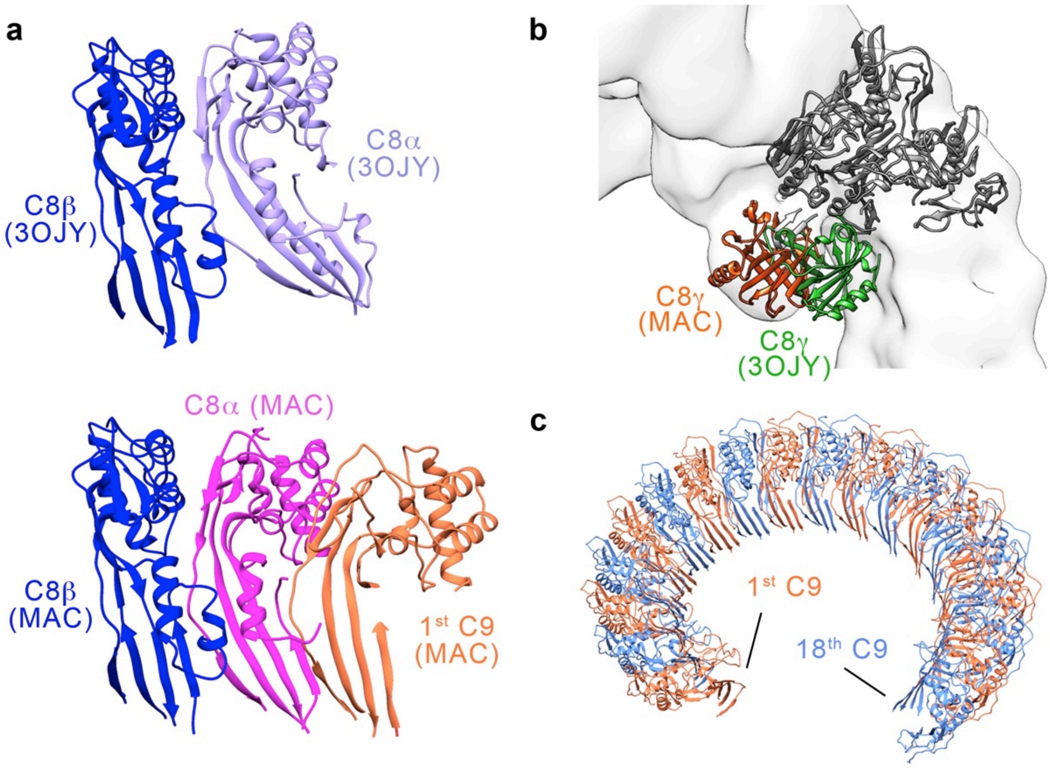

Supplementary Figure 6| Conformational changes of C8 upon integration into the MAC. Structures of C8 within the MAC and its fluid-phase form (PDB ID: 3OJY) were superposed based on the C8β MACPF. The resulting position C8α MACPF **(a)** and C8γ **(b)** was compared. The adjacent C9 MACPF is shown in panel A. A silhouette of the MAC density map is shown for reference in panel B. **(c)** Propagated C9 oligomer, alternating molecules are orange and blue.

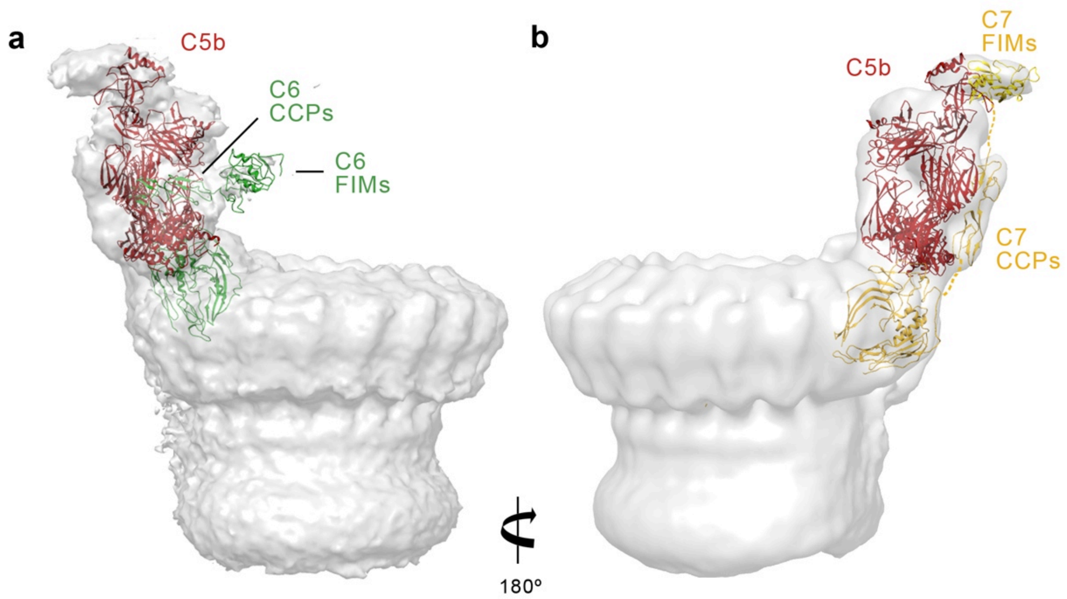

Supplementary Figure 7| C-terminal domains of C6 and C7 within the MAC. (a) Density for the unsharpened MAC reconstruction unfiltered and without mask to show the less ordered regions at a low contour level. Fitting of the C5b6 crystal structure (PDB ID: 4A5W) is shown in ribbons, where C5b is red and C6 is green. **(b)** Fitting of C7 FIMs (PDB ID: 2WCY) and remaining C7 homology model (gold) into the unsharpened MAC map filtered to 12 Å (grey surface). C5b (red ribbons) is shown for reference. Dotted lines connect N- and C- termini of discontinuous chains.

a

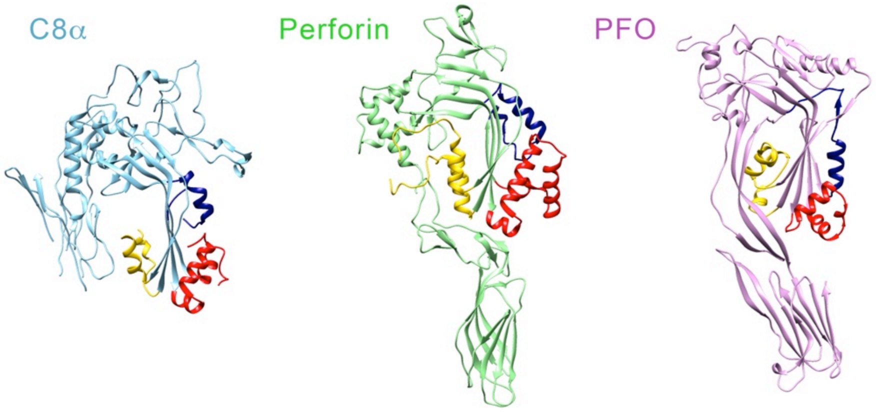

b

CH1

```
C6    (235-289) KDLTSLGHNENQQGSFSSQGGSSFSVPI-------------------FYSSKRSENI-NHNSAFKAIQASHKKD
C7    (180-225) NSTWSYVKHTSTEHTSSSRKRSFFR----------------------------SSSSSSRSYTSHTNEIHKGKS
C8α   (201-256) DNANDLLSKVKKDKSDSFGVTIGIGPAGS------------------PLLVGVGVSHSQDTSFLNELNKYNEKK
C8β   (181-234) ESYSDFERNVTEKMASKSGFSFGFKIPG-------------------IFELGISSQSDRGKHYIRRTKRFSHTK
C9    (197-270) EEQIEAFKSIIQEKTSNFNAAISLKFTPTETNKAEQCCEETASSISLHGKGSFRFSYSKNETYQLFLSYSSKKE
```

CH2

```
C6    (362-414) SEELKNSGLTEEEAKHCVRIETKKRVLF----AKKTKVEHRCTTNKLSEKHEGSFIQ
C7    (299-346) SEKLKQNDFNSVEEKKCKSSGWHFVVK---------FSSHGCKELENALKAASGTQN
C8α   (329-385) KAKMESLGITSRDITTCFGGSLGIQYEDK-INVGGGLSGDHCKKFGGGKTERARKAM
C8β   (308-365) KEAMERGDYTLNNVHACAKNDFKIGGAIEEVYVSLGVSVGKCRGILNEIKDRNKRDT
C9    (343-400) KASMKRKGVELKDIKRCLGYHLDVSLAFSEISVGAEFNKDDCVKRGEGRAVNITSEN
```

Supplementary Figure 8| Structural and sequence homology of predicted transmembrane segments. (a) Crystal structures of C8α (PDB ID: 3OJY) in blue, perforin (PDB ID: 3NSJ) in green, and perfringolysin O (PDB ID: 1PFO) in pink are shown. CH1, CH2, and CH3 helical regions within the CDC/MACPF domain are colored yellow, orange, and blue, respectively. **(b)** Sequence alignment of residues within CH1 and CH2 of MAC component proteins.

Supplementary Table 1| Statistics of model refinement

Rigid Body	Composition	PDB ID	Homology Model*	Map	Fitting (CC)
C5b6	C5b (without C345C) + C6 (without TS1 and FIMs)	4A5W	-	Unsharpened MAC	0.92
C7[1]	C7 (TSs + LR + MACPF + EGF)	-	Based on C6 (PDB ID: 3T5O)	Unsharpened MAC	0.88
C7[2]	C7 (CCPs)	-	Based on C6 (PDB ID: 3T5O)	Unsharpened MAC	0.94
C8β[1]	C8β (TSs + LR + linchpin† + EGF)	3OJY	-	Sharpened MAC	0.87
C8β[2]	C8β (MACPF)	3OJY	-	Sharpened MAC	0.86
C8α[1]	C8α (TSs + LR + linchpin† + EGF)	3OJY	-	Sharpened MAC	0.87
C8α[2]	C8α (MACPF)	3OJY	-	Sharpened MAC	0.85
C8γ	C8γ	3OJY	-	Sharpened MAC	0.91
C9[1]	C9 (TS +MACPF + EGF)	-	Based on C8α (PDB ID: 3OJY)	Sharpened barrel	0.87 ± 0.02‡
C9[2]	C9 (LR)	-	Based on C8α (PDB ID: 3OJY)	Sharpened barrel	0.91 ± 0.01‡

* I-TASSER on-line server (http://zhanglab.ccmb.med.umich.edu/I-TASSER)
† Linchpin helix: C8α MACPF residues A448-E463, C8β MACPF residues S429-E445.
‡ Average correlation coefficient value over 16 subunits.

Supplementary Table 2| Mass spectrometry of the MAC

Protein Accession*	Protein Description	Protein Score†	% Sequence Coverage‡
P01031	Human complement component C5	2843.504	54.3
P13671	Human complement component C6	835.0361	30.62
P10643	Human complement component C7	1394.01	25.39
P07357	Human complement component C8 alpha chain	1019.504	28.42
P07358	Human complement component C8 beta chain	485.4781	23.69
P07360	Human complement component C8 gamma chain	9337.609	47.52
P07357	Human complement component C9	9319.673	38.52

* Protein Accession number for the UniProtKB database used for protein identification by ProteinLynx Global SERVER™ (Waters)
† Protein Scores are assigned by the ProteinLynx Global SERVER™ software to identify the top ranked proteins. The score is based on the peptide precursor tolerance, the number of identified *b* and *y* ions that meet the defined tolerance and ranked based on the sum of the matched *b* and *y* ion intensities.
‡ Percentage sequence coverage indicates the regions of the protein sequence covered by supporting peptide *b* and *y* ions.
